# Stress and suicidal ideation in Korean baby boomers: the mediating effect of mindfulness and meaning in life

**DOI:** 10.3389/fpsyg.2023.1215541

**Published:** 2023-08-28

**Authors:** Yusoo Jeong

**Affiliations:** Department of Psychology, Jeonbuk University, Jeonju-si, Jeollabuk-do, Republic of Korea

**Keywords:** baby boomer, stress, suicidal ideation, mindfulness, meaning in life

## Abstract

Stress has been shown to enhance elderly suicidal ideation. However, the effect of mindfulness and meaning in life on the relationship between stress and suicidal ideation in Korean baby boomers are still unknown. This study investigated (a) a significant correlation between the stress, suicidal ideation, mindfulness, and meaning in life, (b) the mediating effect of mindfulness in the association between stress and suicidal ideation, and (c) the serial mediating effect of mindfulness and then meaning in life on the relationship between stress and suicidal ideation. Data were collected from 200 baby boomers (born between 1955–1963) concerning their stress, mindfulness, meaning in life, and suicidal ideation. These findings suggest that if interventions directed at baby boomers can successfully improve their mindfulness and by extension raise their meaning in life, suicidal ideation will decrease within their population.

## 1. Introduction

Korea maintains the highest suicide rate among OECD countries. The number of suicide deaths in 2021 was 13,352, reflecting an unfortunate increase of 157 (1.2%) over the previous year, while the suicide rate (the number of suicides per 100,000 persons) also increased by 0.3 (1.2%) to 26.0 (KOSIS, 2022). Although it is difficult to definitively identify the cause of this increase, depression and increased suicidal ideation due to stress caused by COVID-19 likely played a role (MOHW, 2022). The suicide rates of those aged 60 and over were 28.4 (per 100,000) among those in their 60s, 41.8 among those in their 70s, and 61.3 among those in their 80s and older, all high compared to the OECD averages (15.2 in their 60s, 16.4 in their 70s, and 21.5 in their 80s and older) (KOSIS, 2022). In light of this, it is urgent to prepare measures that reduce the suicide rate among the elderly generation. The baby boom generation in Korea is comprised of those born between 1955 and 1963. This generation accounts for around 14.5% of the population (about 7.27 million people) (KOSIS, 2018). Ten years ago, the suicide rate of this was 39.1 (per 100,000 people) (KOSIS, 2011), but by 2019, the suicide rate had surged to 46.6 (MOHW, 2021). This was also much higher than the OECD average of 17.2. The baby boom generation is known to have different stressors than the other older generations, distinct from the stress associated with the processes of aging such as deterioration of physical function. Baby boomers are particularly saddened when they can no longer make a productive contribution to society in their old age (Kim, 2018). Moreover, their lack of preparation for old age is a stressor, as this generation had the burden of providing both parental and childcare (Ham and Nam, 2018). For these reasons, the suicide rate among the baby boom generation is considered to be higher than that of previous generations (Kim, 2021). Reducing suicidal ideation is important for the healthy old age of baby boomers. Therefore, the current study investigated the association between stress and suicidal ideation of Korean baby boomers, as well as the influence of mindfulness and meaning in life on this relationship.

### 1.1. The relationship between stress and suicidal ideation

Stress is a strong predictor of suicide (Dixon et al., 1992; Yang and Clum, 1994; Wang et al., 2007). Suicidal behavior can be caused by the interaction between various factors (Wilcox et al., 2010), one (particularly well-documented empirically over the past 40 years) being stressed (Joiner, 2005; Wenzel and Beck, 2008; Hawton et al., 2012; Stewart et al., 2019; Howarth et al., 2020). According to prior studies, those who attempted suicide reported higher levels of stress than those who did not attempt suicide despite feeling depressed (Paykel et al., 1975; Khan et al., 2008; Farahbakhsh et al., 2020). As previous studies have revealed, stress affects suicidal ideation (Howarth et al., 2020). Suicidal ideation can be defined as thoughts about suicide (White, 1989; Beck and Steer, 1991; O’Carroll et al., 1996; Liu et al., 2020), intent to commit suicide, thoughts about committing suicide, and individual wishes to die (McAuliffe, 2002). Suicidal ideation has been shown to increase the risk of actual suicide and is a precursor to suicide attempt or death by suicide (Borges et al., 2006; Yates et al., 2019). Because suicidal ideations are more common than suicide attempts (Fergusson et al., 2003), interventions for individuals experiencing this offer an opportunity to intervene and prevent suicide (Woosley et al., 2014; Walsh et al., 2022).

Stress is defined as harmful life events that affect an individual’s psychological and physiological adaptation (Holmes and Rahe, 1967). Elderly individuals experience health decline, financial difficulties, bereavement, intergenerational conflicts, and family estrangement during their old age, which function as stressors that jeopardize their mental well-being (Moos et al., 2006; Kim, 2009). The experience of stress caused by these issues has been reported to be closely associated with mental health problems such as depression (Bailly et al., 2012). In particular, the elderly experience losses such as the death of friends or spouses, and these experiences of loss have been proposed as stressors that trigger depression in the elderly (Bae, 2009; An and Park, 2016).

Although stress has a negative impact, it is not necessarily the case that everyone who experiences stress will also experience suicidal ideation. The experience of stress is determined by the subjective perception of the person experiencing it, rather than the objective risk or significance of the event (Lazarus and Folkman, 1984; Han, 2008). Wenzel and Beck (2008) revealed that cognitive factors individuals possess play a specific role in the relationship between stress and suicidal ideation. Some individuals at risk for suicide exhibit cognitive distortions (such as dichotomous thinking, jumping to conclusions, and magnification) even when they are not currently experiencing psychiatric symptoms or a suicidal crisis. These maladaptive cognitive styles create chronic patterns of cognition that worsen distress in life stress situations, thereby increasing the likelihood of progressing to suicidal attempts (Wenzel and Beck, 2008). In Korea, it has been demonstrated in research that maladaptive cognitive styles have an impact on the relationship between stress and suicidal ideations (Lee and Lee, 2009; Ryu, 2010).

Through the Mindsponge theory, we can gain a deeper understanding of the cognitive mechanisms underlying suicidal ideation (Vuong et al., 2022). The process of information processing in the mind is complex and dynamic, but it can be summarized by the following five main principles. (a) An information particle must exist within a mind (the subjective world) to be processed by the mind. (b) The information processing mechanism within the mind (the multi-filtering process, or absorption and ejection processes of information and values) is based on the trust evaluator and subjective cost–benefit judgment to maximize the perceived benefits and minimize perceived costs. (c) The multi-filtering process depends on the value system shaped by the mindset (a set of core values). (d) The outputs of conscious and subconscious mental processes (e.g., value system, ideas, thoughts, feelings, behaviors, etc.) are influenced by the values within the mind (mainly by the core values in the mindset). (e) An information particle needs to exist in the environment (objective world) and locate within the perceivable range to be absorbed into the mind (subjective world).

Based on these principles, when the Baby Boom generation possesses information particles such as a sense of loss of significance due to economic disengagement, perceiving themselves as burdens to their families due to inadequate retirement preparations, and experiencing feelings of loneliness resulting from family disconnection, stressors easily infiltrate their minds, influencing the values within their minds, and subsequently leading to the emergence of suicidal ideation. Therefore, when examining the relationship between stress and suicidal ideation, a prior investigation into the negative information particles commonly held by the Baby Boom generation is necessary. Furthermore, there is a need to develop intervention strategies that prevent stressors from accessing negative information particles or facilitate the acquisition of more positive information particles.

### 1.2. The mediating effect of mindfulness on the relationship between stress and suicidal ideation

Mindfulness, the first parameter, is defined as accepting and noticing all phenomena that occur in oneself and the world as they are by paying attention to them the moment, they occur (Kabat-Zinn et al., 1985). Mindfulness interrupts automatic thought processes that lead to negative emotions and encourages a non-judgmental perspective towards distressing thoughts. As a result, it helps individuals to recognize that emerging distressing thoughts are not actual realities but merely thoughts (Williams et al., 2000). Mindfulness affects the process of attention that contributes to emotional distress and maladaptive behavior (Bishop et al., 2004).

The mind is an information collection-*cum*-processor that can be conditionally updated based on environmental conditions and psychological states (Vuong et al., 2022). Stressors experienced by baby boomers during old age can trigger suicidal ideation when they encounter negative information that aligns with their existing perceptions. Specifically, feelings of inadequacy in preparing for old age due to retirement induced loss of social roles, the burden of dual caregiving responsibilities, and stress arising from family conflicts can lead individuals to perceive themselves as worthless, thereby contributing to suicidal ideation. In this context, mindfulness is expected to reduce the frequency of suicidal ideation in baby boomers by promoting a non-judgmental state of mind, thus preventing incoming negative information from interacting with pre-existing information in their minds.

The effectiveness of mindfulness in reducing stress has been validated through various research studies (Yoon, 2014; Gotink et al., 2015). Based on previous research, we can expect that mindfulness will also have an impact on stress reduction among the elderly population (Jeun and Son, 2012; Lee et al., 2021; Kim and Kim, 2023). Specifically, mindfulness plays a role in inducing self-awareness and recognition of one’s inner experiences, thereby preventing cognitive distortions (Park, 2006). Mindfulness is implicated in the ability to control emotions to maintain mental health (Hayes and Feldman, 2004; Guendelman et al., 2017) and has been shown to be highly effective in alleviating depression and anxiety symptoms (Hofmann et al., 2010; Strohmaier, 2020; Strohmaier et al., 2021). As such, mindfulness has been reported to have an excellent effect on the various physical and mental symptoms associated with stress as well as stress itself (Baer et al., 2006; Kallapiran et al., 2015; Potes et al., 2018).

A systematic literature review of mindfulness studies conducted on the elderly suggests that mindfulness is effective at relieving depression, improving sleep quality, and reducing anger in the elderly (Jeon and Kim, 2022). Mindfulness can also significantly reduce suicidal ideation in the elderly (Kwon and Park, 2016; Lee, 2022). Based on these previous studies, we sought to examine whether mindfulness reduced the suicidal ideation of baby boomers as a first step towards a new intervention to address suicidal ideation in this group.

### 1.3. The mediating effect of meaning in life on the relationship between stress and suicidal ideation

Meaning in life, the second parameter, is defined as having an unconditional meaning in any situation and finding meaning in life, being satisfied with one’s own life, and realizing the true essence and value of human beings (Frankl, 1963). Frankl (1969) proposed experiential value as a way to discover the meaning of human life. This refers to loving someone as a unique existence. Frankl (1969) said that the greatest experience is love, and people can help each other realize their potential by understanding what their loved ones can do and what they should become. From this perspective, it can be understood that family holds an important value in the meaning of an elderly person’s life. In fact, for some people, family is a vital meaning in life. Some patients prioritize the well-being of their families over their own lives and sacrifice themselves (Vuong et al., 2023). Disconnection with parents can also increase the likelihood of suicidal ideation (Logan et al., 2011). Thus, when elderly people cannot find value in their relationships with loved ones, their lives may be in greater crisis. In fact, according to previous research, the elderly often give up on life by forgoing interactions with family members (Fitzpatrick and Kim, 2008). Even for elderly patients admitted to intensive care units, the presence of family members has been shown to have a significant impact on their recovery (Olsen et al., 2009). These results show that the influence of family on the meaning of life or death for the elderly is considerable.

Meaning in life plays a very important function in overcoming crises and difficulties. When meaning in life is low level, suicidal ideation, and drug use increases (Harlow et al., 1986; Heisel et al., 2016), leading to negative and dangerous outcomes such as depression and suicide (Bamonti et al., 2016; Neimeyer and Sands, 2017). Individuals who experience lethargy and emptiness due to loss of meaning in life may take a passive attitude or turn to undesirable solutions in lieu of trying to overcome a negative situation (Jang and Hyun, 2016). It has also been shown that drug and alcohol addiction, violence, impulsive behavior, other abnormal behavior, and suicide are all considered in stressful situations (Triplett et al., 2012; Park and Gutierrez, 2013; Liu et al., 2021). When an elderly person is unable to find the meaning in life in their old age, they can regret decisions made in the past and spend the last years of their lives not satisfied with reality (Wrosch et al., 2004). In this manner, the loss of meaning creates a psychological state that leads to suicide (Kim and Kwon, 2012; Nam et al., 2019).

On the other hand, when an elderly person has a strong sense of meaning in life, they can gain self-control and confidence (Krause and Shaw, 2003; Lee and Ha, 2020). Such persons can exercise control by giving a meaningful positive interpretation of the situation even in the event of an unexpected situation. These persons can have confidence in themselves by actively responding to the incident (Skaggs and Barron, 2006; Park and Kwon, 2012). In this manner, the meaning in life can act as a protective factor against suicide by promoting adaptive coping methods in response to stress, adversity, and crisis (Park and Folkman, 1997; Kim and Kwon, 2012).

The Mindsponge theory offers valuable insights into understanding the mechanism of suicidal ideation and its potential role in mediating the relationship between the meaning in life and stress related suicidal ideation (Nguyen et al., 2021). The theory posits that individuals accept or reject new values based on the context they encounter. Individuals experiencing suicidal ideation contemplate suicide as an option when faced with specific circumstances, such as depression, anxiety, perceived burden, and loneliness. This consideration of suicide becomes one of the available alternatives, alongside seeking help or engaging in more meaningful activities to address their challenges. When information about suicide successfully enters an individual’s mind and becomes one of their core preferences, it triggers the emergence of suicidal ideation. In other words, suicidal ideation forms when individuals perceive suicide as a potentially beneficial choice.

Severe mental and physical distress may elevate this suffering to a perceived cost of life, leading individuals to view suicide as an advantageous solution. However, the perceived cost of life due to distress diminishes when individuals find effective sources of assistance. In this context, effective sources of help can be activities involving the discovery and pursuit of the meaning in life. When such assistance is present and accessible, individuals prefer not to accept but instead discard information related to suicide.

Thus, elevating the level of meaning in life for elderly individuals can potentially act as a filter for suicidal information arising from various stressors, thereby reducing suicidal ideation. By nurturing a stronger sense of purpose in life among the elderly, one can mitigate the impact of stressors and effectively diminish the likelihood of suicidal ideations stemming from diverse stressors.

In old age, the meaning in life has a greater impact on health and happiness than in other generations (Bamonti et al., 2016) and reduces suicidal ideation (Linehan et al., 1983; Kim and Kwon, 2012; Nam et al., 2019). This suggests that research on the meaning in life needs to be actively conducted to reduce the high elderly suicide rate in Korea. Therefore, in this study, we attempted to examine the impact of a strong sense of meaning in life on the ability to overcome psychological difficulties in baby boomers and develop suggestions that would improve their quality of life.

This study examines the relationship between variables by setting mindfulness and the meaning in life as mediators, i.e., psychological mechanisms that reduce stress and cognitive distortions and thereby reduce suicidal ideations. Mindfulness has been consistently proven to reduce stress levels (Gotink et al., 2015; Jin, 2020), and the meaning in life have also been reported as having a mediating effect on reducing stress (Choi and Kang, 2021). However, to date no study has examined the effects of these two variables on the relationship between stress and suicidal ideation in Korean baby boomers.

### 1.4. Hypotheses of this study

**Figure 1:**
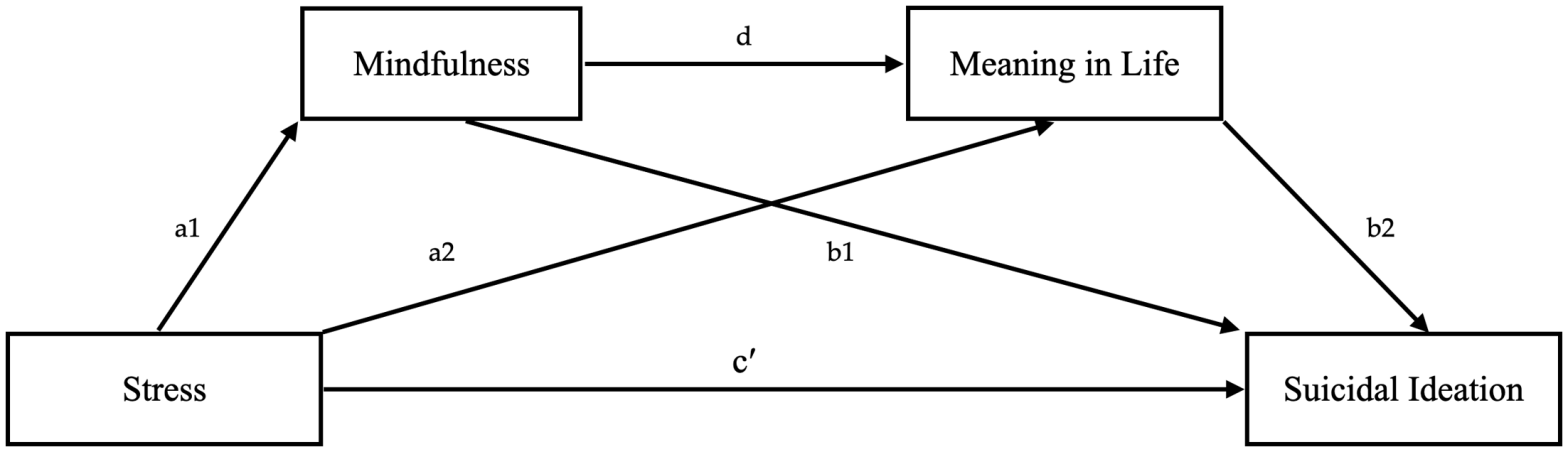
The proposed mediation model.

In this study, a serial mediation model (Figure 1) was proposed to test the mediating effect of mindfulness and meaning in life in the association between stress and suicidal ideation among Korean baby boomers. Specifically, four hypotheses (direct and indirect effects) were examined, as follows:

*H1:* Stress is directly associated with suicidal ideation.

*H2:* Stress is indirectly associated with suicidal ideation via mindfulness.

*H3:* Stress is indirectly associated with suicidal ideation via meaning in life.

*H4:* Stress is indirectly associated with suicidal ideation by mindfulness and then meaning in life.

## 2. Materials and methods

### 2.1. Participants and procedure

Data collection was performed by INVIGHT PANEL Co., Ltd., a panel data collection company, to collect responses to a survey filled out by baby boomers (born between 1955–1963) living all over Korea. Two hundred participants (100 men and women each) were examined. The survey, which collected data on demographic characteristics, stress, mindfulness, meaning in life, and suicidal ideation, was conducted online. The data from the 200 filled out questionnaires were used for the analysis.

The demographic and sociological characteristics of the participants and the levels of the major variables were as follows: Average age was 63.4 (SD = 2.50); 100 participants were male (50%) and 100 were female (50%); 191 (95.5%) were married, 9 (4.5%) were unmarried; and 84 (42.0%) were retired, 116 (58.0%) were not retired. Finally, 130 (65.0%) were college graduates, 36 (18.0%) were high school graduates or lower, and 34 (17.0%) were graduate school or higher.

Participants’ clinical burden is as follows: The degree of stress of the participants was 48.52 (SD = 15.39) and the degree of suicidal ideation was 7.70 (SD = 3.60).

The mean for suicidal ideation is as follows: Male mean 7.82 (SD = 3.62) and Female mean 7.59 (SD = 3.59). The results indicate no significant difference between genders.

This study was approved by the Jeonbuk National University Institutional Review Board (JBNU 2023-01-001-002), and all research procedures were conducted ethically. Data were collected anonymously from all the participants.

### 2.2. Measures

#### 2.2.1. Family inventory of life and events and changes

Stress was measured with the same elderly stress scale used in Lee and Kim (1999). This scale was first developed and used by Kang and Kim (1990) and was based on family Inventory of life and Events and changes (FILE) as modified and supplemented by Lee and Kim (1999). This scale assesses stress levels experienced over the course of the past year. This scale consists of 21 items and five sub-factors: family (nine items, e.g., I have conflicts with my family), economic problem (four items, e.g., I need money for hospital bills and medicine), loss problem: the loss resulting from the death of family members and friends (three items, e.g., I felt destressed and experienced stress due to the death of a close relative), health problem (three items, e.g., I have been hurt or am very sick) and housing problem (two items, e.g., I feel uncomfortable with the structure of my current house). Each item is rated on a five-point Likert scale ranging from 1 (receive none at all) to 5 (receive a lot). This scale ranges from 21 to 105 points, and the higher the score, the more it means that one is experiencing a high level of life stress. In this study, the internal consistency of the items (Cronbach’s α) was 0.92.

#### 2.2.2. Mindfulness scale

Mindfulness was assessed using a scale developed by Park (2006). This scale consists of 20 items and four sub-factors: a concentration part (five items, e.g., It’s hard to concentrate on one task), de-centered attention part (five items, e.g., I am often unaware of the moment-to-moment changes in my mood), present awareness part (five items, e.g., I am often worried about the misfortunes that may come), and non-judgmental part (five items, e.g., Sometimes I cannot tell what my feelings or emotions are). Each item is rated on a five-point Likert scale ranging from 1 (not at all true) to 5 (very true). The internal consistency of the items (Cronbach’s α) was 0.95.

#### 2.2.3. Meaning in life questionnaire

Meaning in life was measured using a scale developed by Steger (2005) and validated by Won et al. (2005) using the Korean version. This scale consists of ten items and two sub-factors: existence of meaning (five items, e.g., I know very well what makes my life meaningful) and pursuit of meaning (five items, e.g., I’m looking for meaning in my life). Each item is rated on a seven-point Likert scale ranging from 1 (not at all true) to 7 (very true). The internal consistency of the items (Cronbach’s α) was 0.94.

#### 2.2.4. Suicidal ideation scale

Suicidal ideation was measured using a scale developed by Harlow et al. (1986). This scale is a measurement tool that assesses suicidal ideation experienced over the course of the past year. This scale consists of five items (e.g., I’ve told someone I want to die). Each item is rated on a five-point Likert scale ranging from 1 (not at all true) to 5 (very true). In the study conducted by Lee and Cho (2013), a factor analysis using orthogonal rotation was conducted on the items of the scale. The results revealed that all items demonstrated factor loadings of 0.5 or higher, indicating the suitability of the Suicidal Ideation Scale for assessing suicidal thoughts. The total score is 25 points, and a higher score indicates a greater likelihood of experiencing suicidal ideation. The internal consistency of the items (Cronbach’s α) was 0.90.

### 2.3. Statistical analyses

The data were analyzed using IBM SPSS Statistics software for Windows 26.0 and PROCESS Macro 3.5. The skewness and kurtosis of the data were checked using parametric statistical analysis. Pearson’s product–moment correlational analysis was conducted using SPSS and a sequential mediating effect was analyzed using PROCESS Macro 3.5 Model 6 (Hayes et al., 2017). During the serial mediation analysis, the nature of the relationship between X and Y (X: stress and Y: suicidal ideation) was assessed directly. Additionally, the indirect effect resulting from the two mediators mindfulness and meaning in life, as well as their indirect serial mediation effect (Figure 1), were tested. The analytical workflow was based on previous work by Preacher and Hayes (2008), where multiple mediation analysis relies on two elements. First, an examination is made to conclude whether the set of mediators transmits the effect of X to Y, and second, the specific indirect effect associated with each presumed mediator is tested. Within this framework, total indirect effects need not be significant for identification of relevant specific indirect effects.

Total, direct, indirect, and partial effects included in the model were described as statistically significant if the corresponding 95% confidence interval of the unstandardized effect size coefficient b did not contain zero. If the direct path between X and Y (c′) was significant, and all three indirect pathways (a1 × b1; a2 × b2; and a1 × d × b2) yielded significant results, a partial serial mediation model is present. If the c′ path effect between X and Y is non-significant and the three indirect pathways were significant, a full serial mediation model is present. If any of the indirect pathways fail to reach significance, the remaining indirect pathways were examined to assess the model.

During the Macro PROCESS analyses, bootstrap resampling value was set at 5,000. Each of the pathways was tested by regressing the corresponding variables. If the b coefficient of the estimated direct, serial indirect, or independent indirect effects occurred within a 95% confidence interval range excluding zero, the null hypothesis of no significant predictive effect was rejected.

## 3. Results

### 3.1. Pearson’s correlation results

Table 1 presents the correlational analysis of stress, mindfulness, meaning in life, and suicidal ideation in Korean baby boomers. None of the absolute values for skewness and kurtosis exceeded 2 and 7, respectively, indicating that the variances of all variables were close to the normal distribution for a parametric statistical analysis.

**Table 1:** Correlation coefficient of stress, suicidal ideation, mindfulness, and meaning in life (*N* = 200).

Variables	1	2	3	4
1. Stress	–			
2. Suicidal ideation	0.325**	–		
3. Mindfulness	−0.450**	−0.548**	–	
4. Meaning in life	−0.218**	−0.343**	0.395**	–
*M*	48.520	7.700	85.950	47.995
SD	15.399	3.602	12.172	11.304
Skewness	0.606	1.588	−1.934	−0.376
Kurtosis	−0.057	2.097	5.284	−0.009

**p < 0.01.

The correlational analysis revealed that stress (*r* = −0.218, *p* < 0.01) and suicidal ideation (*r* = −0.343, *p* < 0.01) were negatively correlated with meaning in life, and that mindfulness (*r* = 0.395, *p* < 0.01) was positively correlated with meaning in life. Mindfulness was negatively correlated with stress (*r* = −0.450, *p* < 0.01) and suicidal ideation (*r* = −0.548, *p* < 0.01). Suicidal ideation was positively correlated with stress (*r* = 0.325, *p* < 0.01).

### 3.2. Testing for a serial mediation model

**Figure 2:**
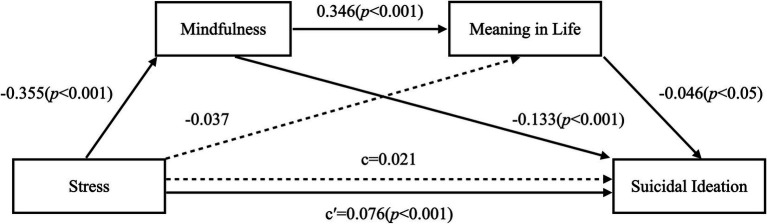
Serial mediation model shows effects of stress, mindfulness, and meaning in life on suicidal ideation.

This study examined the mediating effects of stress, suicidal ideation, mindfulness, and meaning in life in Korean baby boomers (Table 2; Figure 2). Statistical multicollinearity problems are known to occur when tolerance is less than 0.2 or 0.1 and variance inflation factors (VIF) are greater than 5 or 10. As the tolerance of predictors in this study were 0.705 ~ 0.842 and VIFs were 1.188 ~ 1.419, the multicollinearity problem was not significant. Additionally, the value of the Durbin–Watson statistic was 2.026, indicating that there was no autocorrelation detected in the sample.

**Table 2:** Serial mediating effect of mindfulness and meaning in life on stress and suicidal ideation of baby boomers.

Path	*B*	*S.E.*	*t*	LLCI	ULCI
Stress → Mindfulness	−0.355	0.050	−7.091***	−0.454	−0.256
Stress → Meaning in life	−0.037	0.053	−0.695	−0.143	0.068
Mindfulness → Meaning in life	0.346	0.068	5.086***	0.211	0.479
Stress → Suicidal ideation	0.021	0.015	1.391	−0.008	0.051
Mindfulness → Suicidal ideation	−0.133	0.020	−6.424***	−0.173	−0.092
Meaning in life → Suicidal ideation	−0.046	0.020	−2.279*	−0.086	−0.006

*p < 0.05, ***p < 0.001. LLCI: lower level for confidence interval; ULCI: upper level for confidence interval.

Stress was shown to negatively influenced mindfulness (*B* = −0.355, *p* < 0.001), mindfulness was shown to positively influenced meaning in life (*B* = 0.346, *p* < 0.001), and meaning in life was shown to negatively influence suicidal ideation (*B* = −0.046, *p* < 0.05). This confirms the mediating path of mindfulness and meaning in life on stress and the suicidal ideation.

Using 95% bootstrap confidence intervals from 5,000 bootstrap replications, the serial mediating effect of mindfulness and meaning in life on the relationship between stress and suicidal ideation was verified in baby boomers. Results are presented in Table 3.

**Table 3:** Indirect effects of the mediation model.

Path	Effect	*S.E.*	BC 95% CI
Stress → Mindfulness → Suicidal ideation	0.047	0.012	0.0249 ~ 0.0736
Stress → Meaning in life → Suicidal ideation	0.001	0.003	−0.0035 ~ 0.0088
Stress → Mindfulness → Meaning in life → Suicidal ideation	0.005	0.003	0.0003 ~ 0.0131
Total indirect effect	0.054	0.013	0.0309 ~ 0.0826
Direct effect: Stress → Suicidal ideation	0.021	0.015	−0.0089 ~ 0.0517
Total effect	0.076	0.015	0.0451 ~ 0.1071

LLCI: lower level for confidence interval; ULCI: upper level for confidence interval.

The total mediating effect in this model was 0.054 (0.0309 ~ 0.0826), which was significant because there was no 0 between the upper and lower bounds of the bootstrapping at 95% confidence intervals. Verifying the simple mediating effect revealed that the path from stress to suicidal ideation via mindfulness was significant (0.0249 ~ 0.0736). However, the path from stress to suicidal ideation via meaning in life was not significant (−0.0035 ~ 0.0088). The serial mediating effect of mindfulness and meaning in life on stress and suicidal ideation (stress → mindfulness → meaning in life → suicidal ideation) was 0.005 (0.0003 ~ 0.0131), which was significant.

## 4. Discussion

This study explored the relationships between stress, mindfulness, meaning in life, and suicidal ideation in Korean baby boomers, as well as the serial mediating effect of mindfulness and meaning in life on stress and suicidal ideation. This study has produced valuable information that may serve as the basis for further studies and that will be of use to professionals who treat baby boomers with a suicidal ideation.

We found a significant correlation between all variables. Mindfulness and meaning of life showed significant negative correlations with stress and suicidal ideation. These results are consistent with previous studies that stress, and suicidal ideation negatively correlate with mindfulness (Baer et al., 2006; Park, 2010; Kwon and Park, 2016; Jin, 2020; Lee, 2022), and those that have identified a negative correlation with meaning in life (Park and Folkman, 1997; Triplett et al., 2012; Park and Gutierrez, 2013). Both lower stress levels, while lower levels of mindfulness and meaning in life can increase stress levels and suicidal ideation.

We also found a significant mediating effect by mindfulness on suicidal ideation in baby boomers. When the participants experienced a stressful event, a high level of mindfulness was seen to lower the level of suicidal ideation. Consistent mindfulness practices are known to reduce suicidal ideations by helping people avoid negative emotions that can occur through automatic thoughts by viewing thoughts that cause distress with a non-judgmental attitude (Williams et al., 2000).

Because mindfulness helps individuals accept themselves as they are, regardless of their experiences (Park, 2006), it intervenes pre-emptively before cognitive distortions occur in response to stressful events. In this manner, mindfulness can be seen to have a positive effect on the baby boom generation, allowing them to quickly find psychological stability. Specifically, when the baby boom generation faces a stressful event, mindfulness allows them to pay attention to the experience itself without interpreting it while observing their internal and external experiences (Choi and Byun, 2017). Based on this, suicidal ideations are likely to decrease if the baby boom generation, whose lives become more stressful as they age, are trained to notice, and accept everything they experience, in lieu of being encouraged to try to avoid stress.

The mediating effect of meaning in life was found to be insignificant in mediating the relationship between stress and suicidal ideation. In short, when the baby boom generation faces stressful events, even if they result in a strong sense of meaning of life, there is no associated significant reduction in suicidal ideation. Although numerous previous studies have report that meaning in life has a buffering effect on stress (Hong, 2008; Ju et al., 2012; Lim and Jeon, 2012; Jang, 2019; Shin, 2022), studies conducted on elderly people aged 65 years or older (Seo et al., 2014), and college students (Cho, 2007) have not identified such an effect. Thus, this appears to be an inconsistent aspect of suicidality. We found that a strong sense of meaning in life did not reduce suicidal ideation that occurs in response to stress. This suggests that other variables must be explored if suicidal ideation is to be reduced in the baby boomers.

Baby boomers were economically responsible for both their parents and children (Ham and Nam, 2018), and it is highly likely that their sense of meaning is intrinsically wrapped up with their ability to make such economic contributions. It is probable that once they are no longer able to contribute economically post-retirement, their sense of meaning declines. For this population to discover a new meaning, it is necessary to precede intervention that can find value other than economic value. As noted earlier, family is an important source of meaning in life, so interventions that promote communication with family members should be prioritized. Particularly in today’s context, where nuclear families are becoming the norm and relationships with family members can easily become distant, interventions that increase the level of support from families are needed.

Finally, as a result of analysing whether the stress of the baby boomers affects suicidal ideation through mindfulness and meaning in life by setting up a serial mediation model, the indirect effect was found to be significant and the direct effect to be insignificant. In short, mindfulness and meaning in life have a full mediating effect on the relationship between stress and suicidal ideation. Both mindfulness and meaning in life are capable of sequentially effecting stress, which in turn changes levels of suicidal ideation.

These findings support the Mindsponge theory, suggesting that the impact of stress on suicidal ideation is not a direct effect, but rather dependent on whether the information generated by stress is absorbed into an individual’s subjective world through filters (Nguyen et al., 2021). By intervening with mindfulness before various stresses experienced by the baby boomers activate automatic thoughts, individuals can critically assess information and choose alternative paths of seeking and discovering the meaning in life instead of considering suicide as an option, which can help reduce suicidal ideation. Based on a review of previous research, it can be observed that mindfulness enhances self-awareness and reduces automatic conditioning, facilitating a more profound discovery of personal meaning (Young, 2016). Mindfulness also enhances positive emotions by expanding attention to them by encouraging metacognitive awareness and positive reappraisal. This also ultimately enables one to develop a sense of meaning in life (Garland et al., 2015).

The defining characteristic of the baby boomer generation is their high contribution to the growth of Korean society in terms of productivity, surpassing other generations. However, the prospect of entering old age for this generation may evoke thoughts of an unhappy and isolated retirement due to the loss of social roles and feelings of loneliness and depression (Kim, 2018). Baby boomers who hold the belief that they become useless entities when they are unable to make economic contributions are more likely to experience the activation of this belief after retirement. Without specific interventions, this activation can lead to suicidal ideation and an increased likelihood of choosing suicide as an alternative. Therefore, the findings of this study suggest the need for alternative activities that help baby boomers find meaning in life beyond economic contributions, aiming to prevent suicidal ideation among them. People with a higher level of meaning in life tend to employ more adaptive stress coping strategies even when facing stressful situations (Halama and Bakosova, 2009), and their ability to maintain hope serves as an important protective factor against suicidal ideation among baby boomers who might consider suicide as an alternative (Mascaro and Rosen, 2006).

## 5. Limitations and future directions

This study demonstrates the necessity of interventions aimed at increasing not only mindfulness levels but also the level of meaning in life together to reduce suicidal ideation among the baby boomers. However, before these results can be generalized, several limitations of this study must be acknowledged. First, all data in this study were collected through online surveys, a process which excludes baby boomers not interested in such an approach. Second, only two factors, were considered as mediating the relationship between stress and suicidal ideation; other factors worth exploring in future studies include health conditions, finances, and resulting stress levels. Third, when conducting research on suicide related, it is necessary to screen for a history of suicide attempts. However, in this study, screening was not conducted. In future studies, if analysis is conducted on participants with a history of suicide attempts, it is believed that it would be helpful in establishing clearer intervention strategies. Fourth, comparing the mediation structures between the baby boomers and the old-old generation would provide a clearer understanding of the specific characteristics of the baby boomers. Conducting future studies targeting the old-old generation is necessary to compare the results with those of this study and gain further insights.

Despite these limitations, this study is significant for its examination of the impact of mindfulness and meaning in life as a possible focus of interventions that will reduce suicidal ideation in baby boomers, who will soon comprise the majority of Korea’s elderly population. The results of this study are helpful for their provision of basic data necessary to formulate policies related to maintaining the mental health of baby boomers in advance of a transformation into a super aged society. Second, while we found that the buffer effect of meaning in life against stress is insignificant, raising the level of meaning in life through mindfulness is significant. This study revealed that the mindfulness and meaning in life variables should be considered together as programs intended to reduce suicidal ideation in baby boomers are developed. Finally, it is meaningful that the suicide prevention measures of baby boomers were examined at a time when the elderly suicide rate is the highest among OECD countries.

## 6. Conclusion

Unfortunately, many elderly people, including baby boomers, choose to commit suicide due to the stress they experience in old age. Korea, which is aging at a very rapid pace, is expected to have the highest aging rate in the world by 2045. From this perspective, the unhappiness of such a large population of elderly people is a serious individual and social problem. The mental health of baby boomers, who account for the largest proportion of Korea’s aging society, must be addressed immediately. Despite the urgency, little research has been conducted on possible interventions that would help reduce suicidal ideation in baby boomers. Since baby boomers tend to define their self-worth in economic terms, they are likely to experience an existential emptiness once they can no longer make a productive financial contribution to society post-retirement. This sense of existential emptiness increases the suicidal ideation. For this reason, it is necessary to help baby boomers redefine and discover a new meaning in life. This study showed that interventions aimed at simply increasing one’s meaning in life do not have the desired effect, but that suicidal ideation can be reduced when efforts to discover a new meaning in life are preceded by efforts to increase mindfulness. Mindfulness reduces negative emotional experiences that occur automatically and allows one to form a foundation for discovering a new meaning in life. In short, the results of this study highlight the need for educational and practical programs designed to increase the mindfulness and foster a new meaning in life to reduce suicidal ideation of baby boomers. As Korea maintains the highest elderly suicide rate among OECD countries, the development and implementation of such programs should be carried out at the national level. This study provides helpful data for the design of such programs.

## Data availability statement

The raw data supporting the conclusions of this article will be made available by the authors, without undue reservation.

## Ethics statement

The study was conducted in accordance with the guidelines of the Declaration of Helsinki and approved by the Institutional Review Board of Jeonbuk National University (protocol code JBNU 2023-01-001-002, January 1, 2023). Written informed consent was not required to participate in this study in accordance with the local legislation and institutional requirements.

## Author contributions

YJ conceived of the study, did the analyses, and interpreted the data, and drafted the manuscript.

## Funding

The research received funding from the Brain Korea 21 Fourth Project of the Korea Research Foundation (Jeonbuk National University, Psychology Department no. 4199990714213).

## Conflict of interest

The author declares that the research was conducted in the absence of any commercial or financial relationships that could be construed as a potential conflict of interest.

## Publisher’s note

All claims expressed in this article are solely those of the authors and do not necessarily represent those of their affiliated organizations, or those of the publisher, the editors and the reviewers. Any product that may be evaluated in this article, or claim that may be made by its manufacturer, is not guaranteed or endorsed by the publisher.
